# Snow Albedo Feedbacks Enhance Snow Impurity‐Induced Radiative Forcing in the Sierra Nevada

**DOI:** 10.1029/2022GL098102

**Published:** 2022-06-03

**Authors:** Huilin Huang, Yun Qian, Cenlin He, Edward H. Bair, Karl Rittger

**Affiliations:** ^1^ Pacific Northwest National Laboratory Atmospheric Sciences and Global Change Division Richland WA USA; ^2^ Research Applications Laboratory National Center for Atmospheric Research Boulder CO USA; ^3^ Earth Research Institute University of California Santa Barbara CA USA; ^4^ Institute for Arctic and Alpine Research University of Colorado Boulder Boulder CO USA

**Keywords:** light‐absorbing aerosols, impurity content in snow, snow albedo change, radiative forcing, snow albedo feedbacks

## Abstract

This study employs a fully coupled meteorology‐chemistry‐snow model to investigate the impacts of light‐absorbing particles (LAPs) on snow darkening in the Sierra Nevada. After comprehensive evaluation with spatially and temporally complete satellite retrievals, the model shows that LAPs in snow reduce snow albedo by 0.013 (0–0.045) in the Sierra Nevada during the ablation season (April‐July), producing a midday mean radiative forcing of 4.5 W m^−2^ which increases to 15–22 W m^−2^ in July. LAPs in snow accelerate snow aging processes and reduce snow cover fraction, which doubles the albedo change and radiative forcing caused by LAPs. The impurity‐induced snow darkening effects decrease snow water equivalent and snow depth by 20 and 70 mm in June in the Sierra Nevada bighorn sheep habitat. The earlier snowmelt reduces root‐zone soil water content by 20%, deteriorating the forage productivity and playing a negative role in the survival of bighorn sheep.

## Key Points

- Light‐absorbing particles decrease snow albedo by up to 0.045 during April–July, producing radiative forcing of up to 22 W m^−2^
- Impurity effects are amplified by a factor of 2–3 due to snow albedo feedbacks associated with snow aging and snow cover change
- Impurity effects decrease snow water equivalent by 20 mm in June and reduce root zone soil water content by 20% in bighorn sheep habitat

## 1 Introduction

The mountain snow located in the Sierra Nevada (SN) receives precipitation during winter and gradually releases melting water through the spring and dry summer, acting as low‐cost water storage. The snowmelt runoff is captured by reservoirs and released to the downstream community, contributing to 60% of the state’s consumptive water supply (Avanzi, 2018). The snowpack also plays a vital role in the survival of the Sierra Nevada bighorn sheep, an endangered subspecies of bighorn sheep living in the mid‐high elevations of the SN. During winter, aboveground snow impedes the travel of bighorn sheep and reduces the forage availability (Conner et al., 2018), while melting snow supplies soil water for plant growth and influences the nutrition level of bighorn sheep in the following spring and summer (Monteith et al., 2014; Stephenson et al., 2020).

The general balance between snowmelt water supply and water demand in the SN is challenged due to decreased peak snow volume and earlier snowmelt in a changing climate (Huning & AghaKouchak, 2018; Kapnick & Hall, 2010; Wanders et al., 2017). Light‐absorbing particles (LAPs) in snow, mainly dust and black carbon (BC), enhance melting by decreasing surface albedo in the visible and near‐infrared, referred to as the “instantaneous snow darkening effects” (Hadley & Kirchstetter, 2012; Hadley et al., 2010; Qian, Wang, et al., 2014; Qian, Yasunari, et al., 2014; Skiles et al., 2018; Warren & Wiscombe, 1980). The darkened snow absorbs more sunlight, accelerating snow aging, increasing snow grain sizes, and exposing more dark surfaces, known as the “snow albedo feedback” which further reduces surface albedo (Myhre et al., 2013). The snow darkening effects have large spatial variability and are influenced by the LAP emissions, transport, and post‐depositional processes (Kang et al., 2020). Over the Himalaya and the Arctic, LAPs in snow are found to have comparable radiative forcing (RF) to greenhouse gases (Flanner et al., 2007). Snowpacks in the SN also receive large amounts of LAPs from local and remote sources. The ambient dust in the atmosphere is produced from sources such as the dry Owens Lake and the Sonoran Desert (Duniway et al., 2019; Reheis, 1997; Reheis & Kihl, 1995) or transported from as far away as the Sahara and Asia (Creamean et al., 2013). Meanwhile, BC emitted from coastal metropolitan regions, forest fire, or originating from Asia can also be carried to the SN by prevailing westerlies (Hadley et al., 2010; Huang et al., 2020).

While impurity effects have been extensively observed and modeled in the Himalaya and the Rocky Mountains (C. Wu et al., 2018; He et al., 2018; Niu et al., 2018; Oaida et al., 2015; Painter et al., 2007, 2010; Qian et al., 2011; Rahimi et al., 2020; Sarangi et al., 2019, 2020; Skiles & Painter, 2017, 2018), their quantification in the SN is lacking. Observational studies have focused on specific areas and reported a wide range of RF induced by impurities in snow. Sterle et al. (2013) reported a BC concentration of 20–429 ng g^−1^ and a dust concentration of 1–44 μg g^−1^ on the snow surface of Mammoth Mountain, which combined contributed to 20–40 W m^−2^ RF during the 2009 ablation season. Airborne observations over Kaweah/Kings river basins showed that LAPs reduced albedo from 0.70 to 0.55 and caused an RF of 0–150 W m^−2^ (Seidel et al., 2016). The LAP effects show large spatial variability; it is therefore difficult to apply the conclusions to other basins or make inferences at the regional scale.

The Weather Research and Forecasting (WRF) model coupled with online chemistry (WRF‐Chem) has been widely used to assess the interactions between aerosols and snow at the regional scale. As a widely used regional climate model, WRF has been shown to capture fine‐scale precipitation and snow features (Chen, Duan, et al., 2019; Chen, Leung, et al., 2019). An early WRF‐Chem study found that BC reduced albedo by 0.01–0.03 and caused an RF of 1–3 W m^−2^ in the SN (Qian et al., 2009). The result was generally confirmed by Hadley et al. (2010), which used observed BC concentrations in the NCAR CCM3_CRM radiation model to calculate RF induced by BC in snow. Neither study considered the effects of dust in snow, which was observed to play a dominant role in the eastern SN. A recent modeling study by L. Wu et al. (2018) moved further to assess the aerosol–snow interactions due to both BC and dust and compared it with other pathways of aerosol effects: aerosol–radiation interactions and aerosol–cloud interactions. Yet the simulated albedo change has not been thoroughly evaluated due to a lack of observations in the SN. The recently developed satellite products, such as the MODIS Snow‐Covered Area and Grain size algorithm (MODSCAG; Painter et al., 2009) and Snow Property Inversion from Remote Sensing (SPIReS; Bair et al., 2020) retrieve snow properties on the regional scale with complete temporal coverage. They provide instantaneous snow darkening effects for the time satellite overpasses the target pixels, which is especially important in regions with rare site measurements. In addition, the instantaneous darkening effects and snow albedo feedbacks associated with snow aging and snow cover change are two important processes influencing LAP’s darkening effects. Their relative contributions have not been well understood in previous studies. Furthermore, there is a lack of process‐level analyses on how snow darkening effects evolve with LAPs depositions, precipitation, snow aging, and meltwater runoff across the SN, which would facilitate a more realistic prediction of snow darkening effects in a changing world.

The objective of this study is: (a) to evaluate the WRF‐Chem representation of snow properties and snow darkening effects against satellite retrievals and elucidate model uncertainties in the SN; (b) to separate and quantify instantaneous darkening effects and the snow albedo feedbacks associated with snow aging and snow cover change; (c) to comprehensively analyze the snow darkening effects caused by LAPs’ deposition, precipitation, snow aging, and meltwater scavenging processes.

## 2 Model and Method

### 2.1 Model and Input Data

We used the state‐of‐the‐art WRF‐Chem version 3.9 coupled with the Community Land Model (CLM4) land surface scheme, and the Snow, Ice, Aerosol, and Radiation model (SNICAR) to study snow evolution in the SN. This coupled model is referred to as WCCS hereafter. In WCCS, we chose the Model of Ozone and Related chemical Tracers (MOZART) chemistry module (Emmons et al., 2020) and the Model for Simulating Aerosol Interactions and Chemistry with four bins (MOSAIC 4‐bin) aerosol model (Zaveri & Peters, 1999) for comprehensive treatment for gas chemistry and aerosol processes (Table S2 in Supporting Infomation S1). The SNICAR simulates the snow properties and associated radiative heating rates of multilayer snowpack (Flanner et al., 2009, 2021) and is used within CLM, which provides the number of snow layers and meltwater transport from each layer (Oleson et al., 2010). Following the methodology in Zhao et al. (2014), we coupled the WRF‐Chem simulated aerosols with CLM‐SNICAR to simulate the aerosol radiative effects in snowpack. Modifications have been made as we used the MOZART in WRF‐Chem v3.9 instead of the CBM‐Z (carbon bond mechanism) in WRF‐Chem v3.5 in Zhao et al. (2014). A detailed description of the coupling strategy is provided in Text S1 of Supporting Information S1.

The meteorological initial and lateral boundary conditions were derived from the ECMWF Reanalysis v5 [ERA5 (Hersbach et al., 2020)] at 0.25° horizontal resolution and 6 hr temporal intervals. Spectral nudging was employed with a timescale of 6 hr above the boundary layer to reduce the drift between ERA5 reanalysis data and WRF’s internal tendencies (von Storch et al., 2000). The MYJ (Mellor–Yamada–Janjic) planetary boundary layer scheme (Hong et al., 2006), Morrison 2‐moment microphysics scheme (Morrison et al., 2009), Grell‐Freitas cumulus scheme (Grell & Freitas, 2014), and RRTMG longwave and shortwave radiation schemes (Iacono et al., 2008) were used in this study.

Anthropogenic emissions provided by the US EPA 2017 National Emissions Inventory (US Environmental Protection Agency, 2021) were updated every hour to account for the diurnal variability. Fire INventory from NCAR version 2.4 (FINNv2.4) provided daily biomass burning emissions for the years 2018 and 2019 (Wiedinmyer et al., 2014). Biogenic emissions were generated using the Model of Emissions of Gases and Aerosols from Nature (MEGAN) (Guenther et al., 2006). Dust emissions were calculated “online” using the GOCART dust scheme (Ginoux et al., 2001), and here, we increased the emission parameter from 1 × 10^−9^ to $5\times 10^{-9}{\;\text{kg}\;m}^{-5}s^{2}\;$ C in Equation S1 in Supporting Information S1 to match the measurements of surface dust concentration (Text S2 in Supporting Information S1). The chemical initial and boundary conditions were provided by CAM‐Chem (Buchholz et al., 2019).

### 2.2 Numerical Experiments

**Figure 1:**
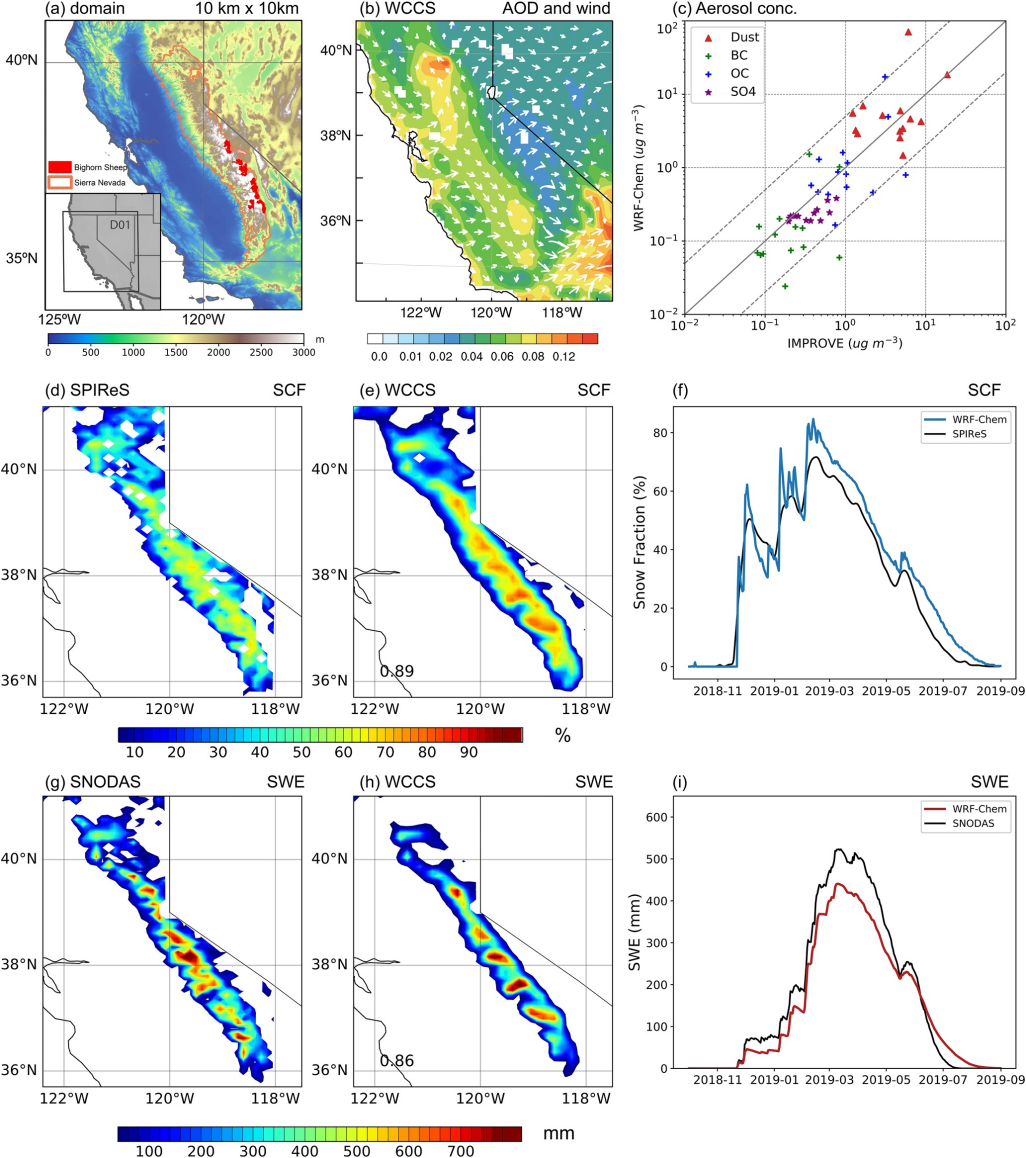
(a) Sierra Nevada region and bighorn sheep habitat, the inserted plot shows WCCS_aero_ simulation domain (D01); (b) modeled AOD with wind vector overlaid; (c) surface concentrations of dust, black carbon (BC), OC, and SO4 in the model compared with IMPROVE observations; (d)–(e) snow cover fraction (SCF) in model and SPIReS; (f) daily SCF in WCCS_aero_ and SPIReS averaged over the Sierra Nevada; (g)–(i) same as (d)–(f) but for snow water equivalent (SWE). Variables in panels (b)–(e) and (g)–(h) are averaged over October 2018 and August 2019.

The WCCS experiment was configured to cover all of California, Nevada, and part of the surrounding states (126.12–112.86°W, 32.3–43.0°N) with 110 × 120 grid cells at 10 × 10 km^2^ horizontal resolution (Figure 1a). We used 35 vertical model layers from the surface to 10 hPa with denser layers at lower altitudes to resolve the planetary boundary layer. The simulation period ranged from 20 September 2018, to 31 August 2019, to allow for the accumulation and ablation of snowpacks in a water year. Only the results after 1 October 2018 were analyzed to minimize the impacts of initial conditions. To quantify the effects of LAPs in snow with and without snow albedo feedbacks, we designed the following two experiments:

1. WCCS_aero_ simulated the direct and indirect effects of aerosols in the atmosphere and the effects of LAPs in snow.
2. WCCS_noaero_ is similar to WCCS_aero_ except that the impurity effects in snow were not included. The deposited LAPs in snow were manually set to 0 in CLM‐SNICAR.

WCCS_aero_ calculated the instantaneous snow albedo reduction and RF caused by LAPs in every model timestep by contrasting the dirty and clean snow albedo under current snow cover, which can be used to evaluate the LAPs’ instantaneous snow darkening effects. The difference between WCCS_aero_ and WCCS_noaero_ showed the changes of snow albedo and RF due to both instantaneous snow darkening effects and snow albedo feedbacks associated with snow aging and snow cover change. The RF refers to the change in net (down minus up) solar irradiance at the surface caused by the impurity effects (instantaneous darkening effects with/without snow albedo feedbacks).

### 2.3 Validation Data

The simulated aerosols from 2018 October to 2019 August were compared against PM2.5 observations from U.S. EPA Outdoor Air Quality Data (US Environmental Protection Agency, 2021) and IMPROVE (Interagency Monitoring of Protected Visual Environments, Access date October 2021 [Malm et al., 1994]), and aerosol surface concentration from IMPROVE measurements. We evaluated the simulated snow water equivalent (SWE) with the SNODAS (Snow Data Assimilation System), which assimilated satellite‐derived, airborne, and ground‐based snow observations into an operational snow accumulation and ablation model (Barrett, 2003), and we evaluated snow cover fraction (SCF) with SPIReS retrievals (Bair et al., 2020). SPIReS was also used to estimate albedo degradation due to LAPs using the difference between modeled clean and observed (dirty) snow albedo. Both SNODAS and SPIReS have been upscaled to the model resolution before comparing them with the simulation results.

## 3 Results

### 3.1 Model Evaluation

Figure 1b shows the spatial distribution of AOD averaged from 2018 October to 2019 August. The AOD maxima in northern California are caused by the emissions during the Camp fire in November 2018, the most destructive and deadliest fire in California history. Additionally, high AOD values are found in the San Joaquin Valley and metropolitan areas such as San Francisco. The urban emissions are transported eastward and are blocked by mountains, producing a minimum AOD on the eastern slope of SN. Meanwhile, high AOD is also shown over southeastern California, due to the dust emissions from the Mojave Desert. The PM2.5 surface concentrations show a similar spatial distribution to AOD (Figure S1 in Supporting Information S1), with slight underestimations compared to EPA and IMPROVE measurements in the Central Valley and overestimations in southern California. The modeled surface aerosol concentrations are generally within a factor of five of the observed concentrations from IMPROVE sites (Figure 1c). After the calibration of the GOCART dust scheme, simulated dust concentration appears unbiased compared to observed dust concentration. Nevertheless, anthropogenic aerosol concentrations (BC, organic carbon, and sulfate) are underestimated compared to the observations, which is a common problem in WRF‐Chem simulations in California and might be related to the coarser model resolution, uncertainties in emission inventory, and/or a lack of important chemical processes in the model (Wang et al., 2020; L. Wu et al., 2017; Zhao et al., 2013).

We evaluate SCF and SWE with multiple observations during the reference period. The heights of mountain peaks increase gradually from north to south (Figure 1a), and correspondingly, large SCFs are found in the southern SN (Figures 1d and 1e). Snowpacks in low elevations (in both northern and southern SN) diminish in April, while snowpacks in the northern SN (>1,500 m elevation) melt out in May, and snowpacks at the top of the southeast SN last until early August. Compared to SPIReS, WCCS_aero_ reproduces the spatial distribution of SCFs with a correlation coefficient of 0.89 and appropriately captures the temporal evolution (Figure 1f). WCCS_aero_ overestimates SCF in the southern part of the SN, which probably comes from precipitation biases due to the coarser terrain representation (not shown). WCCS_aero_ underestimates peak SWE compared to SNODAS (Figures 1g–1i), yet the latter is shown to overestimate SWE in multiple years (Bair et al., 2016). For the 2019 water year, we use April–July as the ablation season to assess the LAP effects in snow in the SN.

### 3.2 Snow Darkening and Radiative Forcing Due To LAP Effects

**Figure 2:**
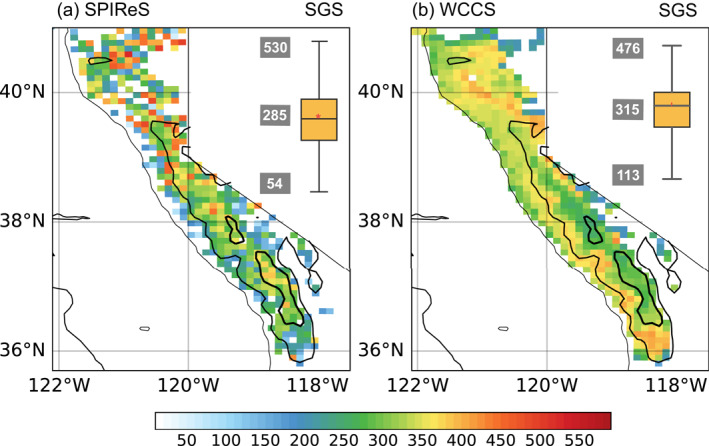
Annual mean snow grain sizes (SGSs) in (a) SPIReS and (b) WCCS_aero_ averaged over 2018 October to 2019 August. The box plots illustrate the distribution of SGSs in SPIReS and WCCS throughout the Sierra Nevada with the mean, maximum and minimum values labeled on the left. The three contours with different thickness represent the elevations of 1,000, 2,000, and 3,000 m.

LAP effects in the snowpack are closely related to the microphysical properties of snowpack, for example, snow grain sizes (SGSs). Annual SGSs from SPIReS range from 54 to 530 μm in the SN, with a regional average of 285 μm (Figure 2a). The regional average SGS in WCCS_aero_ is larger compared to the remotely sensed observations (315 μm) with a smaller spatial variability from 113 to 476 μm (Figure 2b). SGSs are small during winter and rapidly grow in spring because of snow grain metamorphism driven by high temperatures and strong incident irradiance (Flanner & Zender, 2006; Painter et al., 2003, 2013). Larger SGSs are found in the northern SN as warmer temperatures in low elevations facilitate snow aging processes. SGSs at higher elevations (>2,500 m) increase significantly in the late ablation period, producing larger SGSs at the annual time scale in SPIReS (Figure 2a). The model underestimates SGSs growth in the late melting season, therefore producing smaller SGSs at higher elevations as compared to SPIReS (Figure 2b). Retrieved SGSs at lower elevations might be underestimated as these pixels are normally partially covered by snow (SCF <90%) and are interpolated with relatively pure snow pixels (SCF ≥90%) from higher elevations with smaller grain sizes (Bair et al., 2020).

The concentrations of dust and BC on the snow surface range between 4 and 30 μg g^−1^ and 10–40 ng g^−1^ respectively during March‐May, with small variations among different elevations (Figure S2 in Supporting Information S1). Both concentrations increase by a factor of 2–4 as snow melts. In June, the BC concentration decreases with elevation while the dust concentration peaks in both low elevations (1,500–2,000 m) and mid elevations (2,500 m). Our simulated LAPs concentration is generally consistent with Hadley et al. (2010) who reported BC concentrations of 5.3 and 6.9 ng g^−1^ in falling snow during March–April 2006 at two SN locations. At Mammoth Mountain Ski Area, Sterle et al. (2013) found that BC concentration increased from 25 ng g^−1^ during January‐April to 135 ng g^−1^ in May 2009 while dust concentration (12 μg g^−1^) remained relatively stable during the ablation season.

**Figure 3:**
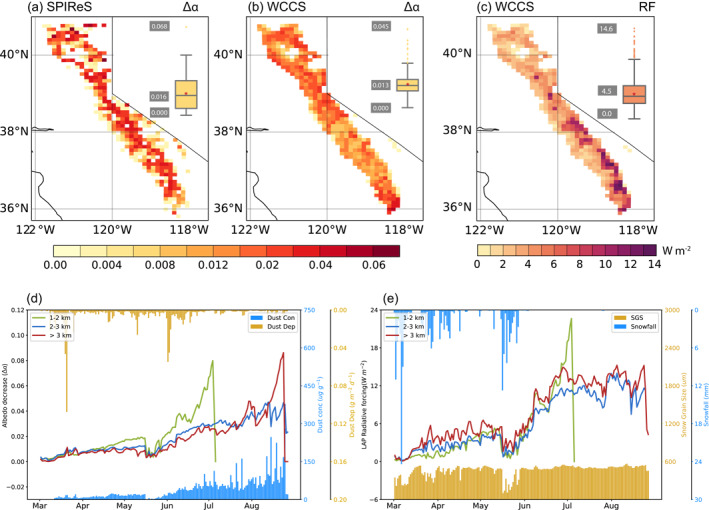
Spatial distribution of snow albedo decrease (∆α) in (a) SPIReS retrievals (b) WCCS_aero_ and (c) RF in WCCS_aero_ during midday (10:00–14:00 LT) averaged over April–July. The box plots illustrate the distribution of ∆α (RF) throughout the Sierra Nevada with the mean, maximum and minimum values labeled on the left; (d) March–August albedo decrease (∆α) during midday (10:00–14:00 LT) at different elevations in WCCS_aero_. The light blue bars show dust concentration on the top snow layer while the yellow bars show dust deposition, averaged over the Sierra Nevada; (e) Simulated RF at different elevations. The yellow bar shows snow grain sizes while the light blue bars show snowfall over the Sierra Nevada.

The dust and BC in snow cause snow darkening which accelerates the snow aging process (Lee & Liou, 2012; Qian, Yasunari, et al., 2014). Satellite retrievals show that the LAP‐induced snow albedo decrease (∆α, larger ∆α represents higher albedo reduction) during April‐July can be as large as 0.068, with an average of 0.016 over the SN (Figure 3a). The ∆α in WCCS_aero_ spans between 0.0 and 0.045, with a spatial average of 0.013 (Figure 3b), slightly lower than the estimate from SPIReS, especially at higher elevations. The estimated ∆α is larger in WCCS_aero_ than SPIReS at lower elevations, while we note that SPIReS might have underestimated ∆α as it interpolates pixels at lower elevations with pure snow pixels (larger albedo) from higher elevations. In both the model and remotely sensed observations, the LAP‐induced ∆α first appears at lower elevations with relatively higher temperatures and thinner snowpacks (Figure S3 in Supporting Information S1). The ∆α in the southern SN higher elevations is small in the early melting season and greatly increases in June and July, corresponding to the later snowmelt there. The model generally captures the spatial distribution of ∆α at the monthly scale but underestimates ∆α in June and July. Throughout the SN, we find LAPs in snow cause an RF of 4.5 W m^−2^ (Figure 3c). The largest RF (14.6 W m^−2^) is found in the southeast SN with higher elevations, despite the lowest AOD there. This is because higher elevations have a later onset of snowmelt and therefore receive higher solar irradiance during the ablation period. The bighorn sheep habitat (Figure 1a red area), with an elevation of 2,840 m on average, is found to have an RF of 12.3 W m^−2^ during the melting season, which can reach 45 W m^−2^ in mid‐June.

Figure 3d shows the evolution of ∆α caused by LAPs in snow at different elevations. The albedo degradation is less than 0.01 during March‐April, which has a steep increase to 0.02 in late May. At lower elevations (1–2 km), the snow darkening effect peaks in late June before snowpacks melt out, while it reaches 0.08 in late August at the higher elevations. The corresponding RF on snow remains generally smaller than 5 W m^−2^ before June and rapidly increases to 15–22 W m^−2^ in July. The contribution from dust is larger in the eastern SN adjacent to the desert, while the contribution from BC is larger in the northwestern SN near the anthropogenic emission and fire emission sources (not shown). Here we focus on dust deposition and accumulation in snow, noting that the BC shows similar changes. The dust concentration on the snow surface varies from 10 to 100 μg g^−1^. During March–May, the deposited dust particles are buried by frequent snowfalls, which add fresh snow with smaller grain sizes to the ground (Figure 3e) and cover surface dust with resulting concentrations at lower levels (Figure 3d). As a result, the LAP‐induced ∆α and RF are small. The abrupt decreases of SGSs after snowfalls were also reported in Seidel et al. (2016) but the magnitude is smaller than our simulation (330–180 μm in observations compared to ∼450 to 100 μm in WCCS_aero_). As snowpacks melt, some dust particles are scavenged away in meltwater but most accumulate, especially at the snow surface (Doherty et al., 2010; Flanner et al., 2007), reflected by the increase of dust concentration by a factor of 4 in the late ablation season. The enrichment of dust concentration exacerbates the increase of ∆α and RF in June and July (Figure 3d).

The underestimation of LAP effects compared to the remotely sensed retrievals can be explained by several aspects. First, WCCS_aero_ underestimates BC surface concentration and might also underestimate BC concentration in snow and BC‐induced LAP effects over the west slope of the SN. Furthermore, the radiative effect of LAPs in snow is amplified with increasing SGSs (Warren & Wiscombe, 1980). The underestimated ∆α at higher elevations in WCCS_aero_ is probably due to insufficient SGSs increases in the late melting season, reflected by the smaller SGSs at the higher elevations compared to SPIReS (Figure 2). Besides, the version of CLM‐SNICAR within WCCS_aero_ does not consider the internal mixing of dust, which is shown to enhance ∆α by 10%–30% for dust relative to the external mixing states (He et al., 2019). Additionally, the structure packing of snow grains is suggested to enhance the LAP‐induced albedo reduction (He, Takano, & Liou, 2017) which is not considered in the model. Conversely, grains are assumed spherical in SNICAR‐CLM, which is not always a correct approximation (He, Takano, Liou, et al., 2017), yet nonspherical snow grains produce smaller radiative effects compared to spherical grains (Dang et al., 2016) and are thus not a cause of underestimated LAP effects. Moreover, SPIReS estimates ∆α based on surface reflectance changes contributed by multiple LAPs [carbon, dust, and algae (Bair et al., 2021)], while our simulation does not consider the effects from brown carbon or snow algae (Kirchstetter et al., 2004; Painter et al., 2001; Thomas & Duval, 1995).

While we use satellite measurements as ground truth, we note that multiple factors may induce the uncertainties in products, including off‐nadir viewing effects, geolocation errors, misclassification between clouds and snow, and errors in the atmospheric model retrieving the aerosol optical depth (Bair et al., 2019, 2020; Stillinger et al., 2019). Extensive validations were conducted against site observations across the western US with RMSE values of 4%–6% in broadband snow albedo between SPIReS and site measurements (Bair et al., 2019). Besides, ∆α from SPIReS is calculated using the difference between observed snow albedo and modeled clean snow albedo; the latter is associated with SGS retrieval, which could also be biased due to the abovementioned uncertainties. In addition, Bair et al. (2020) have made a few assumptions to reduce uncertainties in pixels partially covered by snow: (a) Relatively pure pixels (snow cover fraction >0.90) are used to interpolate SGS to pixels partially covered by snow and therefore produce smaller SGS and (b) Pixels with small SGS (<400 μm) are assumed to be clean. Both assumptions may cause underestimation of LAP‐induced ∆α.

### 3.3 LAP Effect Due To Snow Albedo Feedback

**Figure 4:**
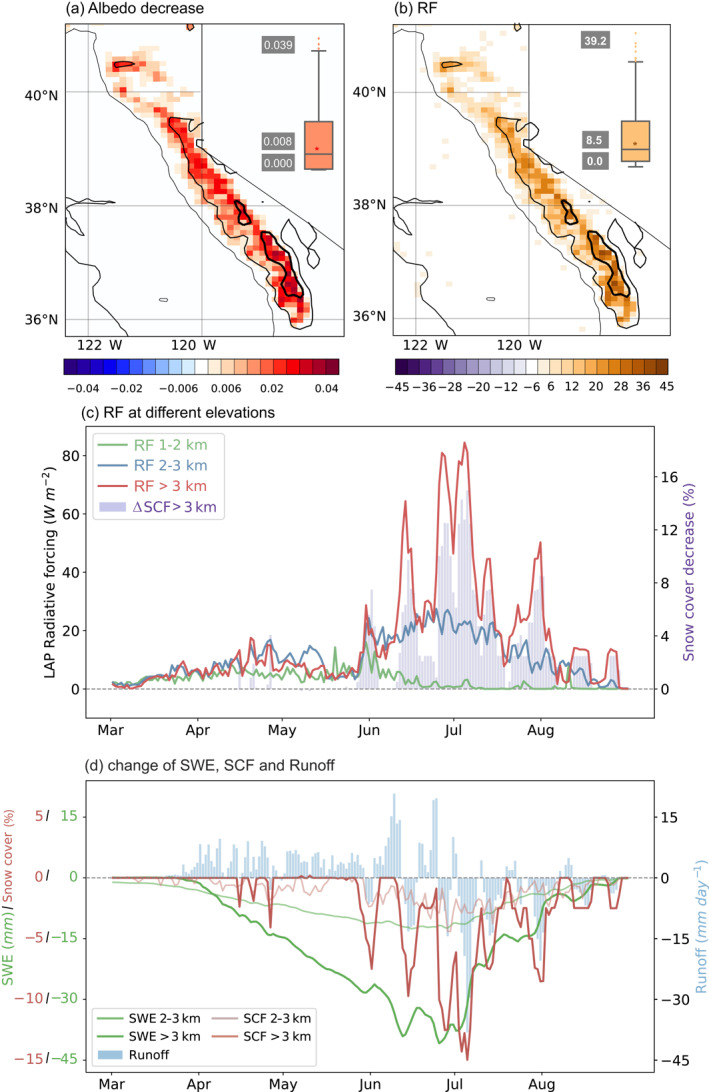
Difference of (a) surface albedo and (b) net shortwave radiation between WCCS_aero_ and WCCS_noaero_ during midday (10:00–14:00 LT) averaged over April–July. The box plots illustrate the distribution of ∆α (RF) throughout the Sierra Nevada with the mean, maximum, and minimum values labeled on the left; The three contours represent the elevations of 1,000, 2,000, and 3,000 m; (c) RF at different elevations and snow cover fraction change. (d) Change of SWE, SCF, and surface runoff between WCCS_aero_ and WCCS_noaero_.

We assess how the snow albedo feedback plays a role in the snow darkening effects by comparing the WCCS_aero_ with WCCS_noaero_. Grid‐averaged surface albedo changes are discussed afterward as snow cover is different in both experiments. The regional albedo decrease is 0.008 averaged across the SN, doubling the value (0.004) we found in LAPs’ instantaneous snow darkening effects (Figure 3b), due to the additional role of snow albedo feedbacks associated with snow aging and snow cover fraction. The albedo reduction is positively correlated with elevation and reaches 0.04 in the southeast SN (Figure 4a). The simulated albedo change is slightly higher than model estimates from L. Wu et al. (2018). The calibrated larger dust emission may produce a larger albedo degradation. Other factors such as different anthropogenic and fire emissions inventories and lateral boundary conditions could also play a role. The albedo change increases solar radiation absorption by about 8.5 W m^−2^ during the midday, with the largest RF reaching 40 W m^−2^ within the high elevations of southern SN (Figure 4b). Averaged over a day, we find an RF of 3.1 W m^−2^ which spans between 0 and 14 W m^−2^. These estimated RF values are higher than those from Qian et al. (2009) and Hadley et al. (2010) which only considered the effects of BC in snow.

LAP‐induced RF increases with elevation, and the peak values occur later at the higher elevations (Figure 4c). The RF is generally smaller than 15 W m^−2^ at low elevations (1,000–2,000 m), with the largest value occurring in late May and disappearing in mid‐June as snowpacks disappear. The RF at mid elevations (2,000–3,000 m) peaks in mid‐June (30 W m^−2^) and lasts until the end of the melting season. The RF at high elevations (>3,000 m) has a similar magnitude to mid elevations during March‐May and significantly increases in June. Several RF peaks are found in June and July, which can be as high as 60–80 W m^−2^ and correspond well with the snow cover reduction (Figure 4c). As discussed before, the albedo changes between clean and dirty snow generally cause an RF of 10–20 W m^−2^ (Figure 3c). The darkened surface absorbs more sunlight which further accelerates snow melting and causes stronger RF. Snow cover losses from this feedback produce an RF of 80 W m^−2^ at the highest elevations, which is a factor of 2–3 greater than the RF due to instantaneous snow darkening effects.

The LAP‐induced RF decreases SWE throughout the melting season (Figure 4d), with the largest reduction (40 mm SWE) in late June. The decrease starts at low elevations (with higher temperatures) and expands to high elevations with later melting season (Figure S4 in Supporting Information S1). The SCF change is small until June and reaches its maximum in earlier July (Figure 4d). Due to LAPs and albedo feedbacks, runoff increases first and decreases in the late melting season. As floods in the Sierra Nevada basins are mainly produced by snowmelt (Huang et al., 2022), the early shift of snowmelt timing is expected to shift snowmelt‐driven peak runoff date to earlier by about 5 days.

In the bighorn sheep habitat, LAP‐induced RF is generally smaller than 2 W m^−2^ during the accumulation season when deep snow impedes travel and negatively influences the bighorn sheep survival (Conner et al., 2018). Consequently, the impact of LAPs in snow on the bighorn sheep is negligible. In the melting season, LAPs in snow have been shown to decrease SWE and snow depth in June by up to 20 and 70 mm, respectively. The decrease in soil water supply reduces root‐zone soil water content by 0.01–0.03 during June‐August (Figure S5 in Supporting Information S1), with the largest reduction found in July. The decreasing soil water content tends to reduce forage productivity in early summer (Liu et al., 2019; Liu et al., 2021; Zhang et al., 2019), which deteriorates bighorn sheep diet quality and nutrition status (Stephenson et al., 2020). The nutrition status, typically measured by body mass, is related to the survival and the reproductive success of bighorn sheep (Festa‐Bianchet et al., 1997).

## 4 Conclusions

The LAPs’ snow darkening effect has been extensively studied in snow‐cover regions, yet the quantification in the Sierra Nevada is rare. This study employs a fully coupled meteorology‐chemistry‐snow model, WCCS, to investigate the impact of LAPs on snow albedo over the Sierra Nevada (SN). Throughout the SN, WCCS approximately reproduces the observed aerosol spatial patterns and realistically simulates the spatial distribution and temporal evolution of snow cover and SWE. The simulation shows that snow albedo is reduced by 0.013 (0–0.045) during the ablation season due to LAPs in snow, producing a radiative forcing of 4.5 W m^−2^ (0–14.6 W m^−2^). Despite the lowest AOD in the southeast SN, the largest RF is found there as the higher elevations receive stronger solar irradiance during the ablation period. The model underestimates snow albedo degradation compared to remotely sensed retrievals (0.016 in SPIReS) at the higher elevations, which may be due to uncertainties in snow impurity concentration, insufficient snow aging process, and unrealistically small grain sizes for new snow.

The darkened snow absorbs more sunlight which accelerates melting and exposure of darker surfaces, leading to the well‐known “snow albedo feedback.” With snow albedo feedbacks, LAPs induce an RF of 8.5 W m^−2^ during the melting season, with the largest RF reaching 80 W m^−2^ in late June. The RF causes a decrease of SWE by 40 mm, shifting the runoff peak earlier.

## Supporting information

Supporting Information S1

## Data Availability

The PM2.5 and aerosol surface measurements are acquired from U.S. EPA at https://www.epa.gov/outdoor-air-quality-data/download-daily-data and IMPROVE sites at http://vista.cira.colostate.edu/Improve/monitoring-site-browser. SNODAS datasets are from the National Snow & Ice Data Center (NSIDC) at https://nsidc.org/data/g02158; SPIReS dataset is available at https://snow.ucsb.edu/index.php/remotely-sensed-products/. We uploaded the data used in this paper on Zenodo: https://doi.org/10.5281/zenodo.5914858.
