# Supplementary material for: Snow Albedo Feedbacks Enhance Snow Impurity‐Induced Radiative Forcing in the Sierra Nevada
Source: Geophys Res Lett. 2022 Jun 3;49(11):e2022GL098102. doi: 10.1029/2022GL098102 (PMC9285762; doi:10.1029/2022GL098102)
Supplement: Supplementary file 1 — Supporting Information S1 [file GRL-49-0-s001.pdf]

Geophysical Research Letter

Supporting Information for

Snow albedo feedbacks enhance snow impurity-induced radiative forcing in the Sierra Nevada

Huilin Huang[1], Yun Qian[1*], Cenlin He[2], Edward Bair[3], and Karl Rittger[4]

[1]Atmospheric Sciences and Global Change Division, Pacific Northwest National Laboratory, Richland, WA, USA

[2]Research Applications Laboratory, National Center for Atmospheric Research, Colorado, CO, USA

[3]Earth Research Institute, University of California, Santa Barbara, CA, USA,

[4]Institute for Arctic and Alpine Research, University of Colorado Boulder, Boulder, CO, USA

*Corresponding to: Yun Qian, Yun.Qian@pnnl.gov; Huilin Huang, Huilin.huang@pnnl.gov

Contents of this file

Text S1. Coupling between WRF-Chem v3.9.1 with CLM-SNICAR

Text S2. Calibration of dust emission to match surface model concentrations from observations

Table S1 to S2

Figures S1 to S3

SI References

Text S1. Coupling between WRF-Chem v3.9.1 with CLM-SNICAR

The CLM-SNICAR was first incorporated in WRF-Chem v3.5 to simulate snow albedo evolution due to snow aging and the presence of aerosols in *Zhao et al.* [2014]. Following their approach, we coupled the WRF-Chem v3.9.1 with CLM-SNICAR to represent the interactions between aerosols and snow in a prognostic approach through dry and wet deposition processes. Modifications have been made in the coupling strategy as *Zhao et al.* [2014] used the CBM-Z (carbon bond mechanism) photochemical mechanism while we used MOZART. Besides, the CMU aqueous phase chemistry [*Fahey and Pandis*, 2001] has been added and used in WRF-Chem v3.9 while it was not used in WRF-Chem 3.5.

The dry deposition of LAPs now includes the diffusion and gravitational effects of aerosols in aerosol and aqueous phases. Wet deposition of LAPs includes in-cloud removal (rainout) and below-cloud removal (washout) by grid-resolved stratiform clouds/precipitation, as well as the sub-grid wet scavenging [*Chapman et al.*, 2009; *Easter et al.*, 2004]. The MOSAIC 4-bin aerosol module simulates dust with size bins of 0.039–0.156, 0.156–0.625, 0.625–2.5, and 2.5–10.0 μm while the SNICAR-CLM simulates dust size of 0.1–1, 1–2.5, 2.5–5, and 5–10 μm. We match the dust size distributions between WRF-Chem and SNICAR-CLM following Table S1 below (reorganized from *Zhao et al.* [2014]).

Table S1 Matching of dust size distributions between WRF-Chem and SNICAR-CLM. The values represent mass fractions of deposited dust from WRF-Chem into each size of dust in snow in CLM-SNICAR.

	SNICAR-CLM			
WRF-Chem	0.1-1	1-2.5	2.5-5	5–10
0.039–0.156	1	0	0	0
0.156–0.625	1	0	0	0
0.625–2.5	0	1	0	0
2.5–10.0	0	0	0.5	0.5

Text S2. Calibration of dust emission to match surface model concentrations from observations

We calculated the dust emissions “online” using the GOCART dust scheme [Ginoux et al., 2001], which is re-distributed into different bins for MOSAIC [Zhao et al., 2010]:

$$F_p = \begin{cases} C \cdot S \cdot s_p \cdot u_{10m}^2 \cdot (u_{10m} - u_t) & (u_{10m} > u_t) \\ 0 & (u_{10m} \le u_t) \end{cases} \qquad \text{(Eq. S1)}$$

where C is an empirical constant, S is the source function, s_p is the fraction of each size class of the emitted dust, u_{10m} is the horizontal wind speed at 10 m and u_t is the threshold wind speed below which dust emissions do not occur. The value of C was initially proposed as 1×10^{-9} kg m^{-5} s^2 but was suggested to be highly tunable [Kumar et al., 2014]. In our study domain, we find the default C (1×10^{-9} kg m^{-5} s^2) produced smaller surface dust concentration as compared to site measurements from IMPROVE (Interagency Monitoring of Protected Visual Environments [*Malm et al.*, 1994]). After sensitivity tests, we used $C = 5 \times 10^{-9}$ kg $m^{-5}s^2$ to match the measurements of surface dust concentration.

Table S2. Model configuration.

Atmospheric processes	WRF-Chem Configuration
Meteorological IC/LBCs	ERA5
Microphysics	Morrison double-moment
Radiation	RRTMG for both shortwave/longwave
Land surface	CLM4 with SNICAR
Surface layer	Revised MM5 Monin-Obukhov
Planetary boundary layer	YSU scheme
Cumulus	Grell-Freitas
Chemical driver	MOZART
Aerosol driver	MOSAIC 4-bin
Anthropogenic emission	NEI2017
Biogenic emission	MEGAN
Biomass burning emission	FINNv2.2
Dust emission	GOCART
Chemical IC/BC conditions	CAM-Chem

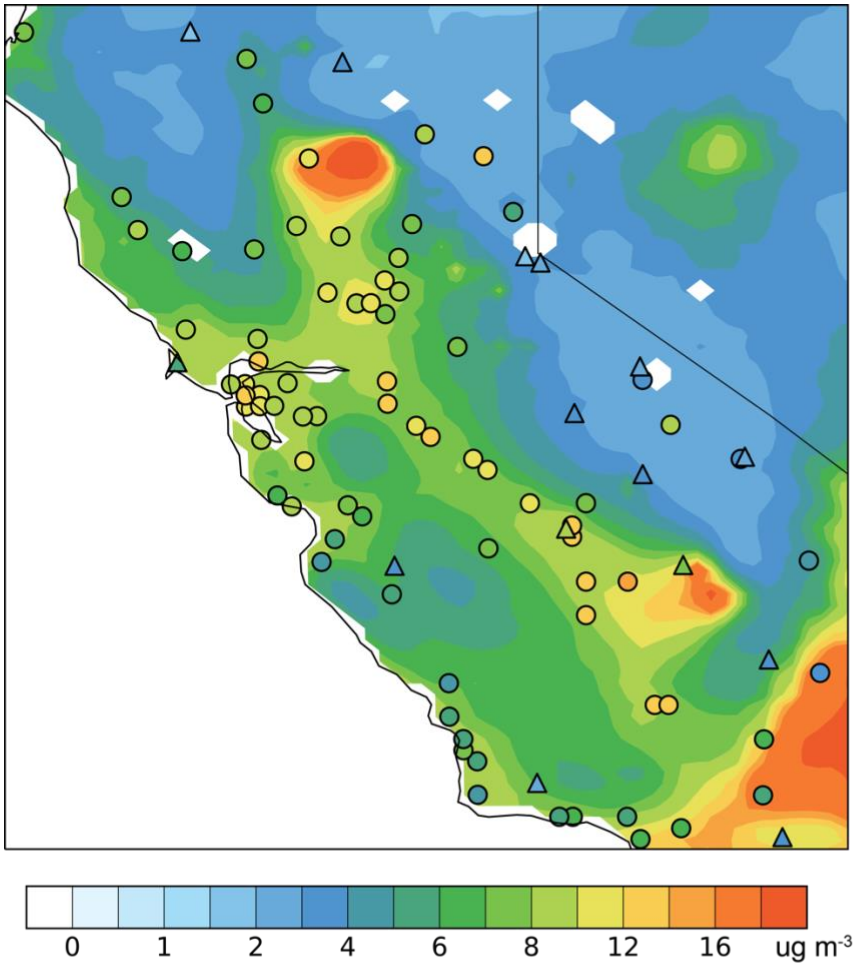

Figure S1 Spatial distribution of PM2.5 surface mass concentrations simulated by $WCCS_{aero}$ averaged over 2018 October to 2019 August compared to observations from EPA (circles) and IMPROVE (triangles)

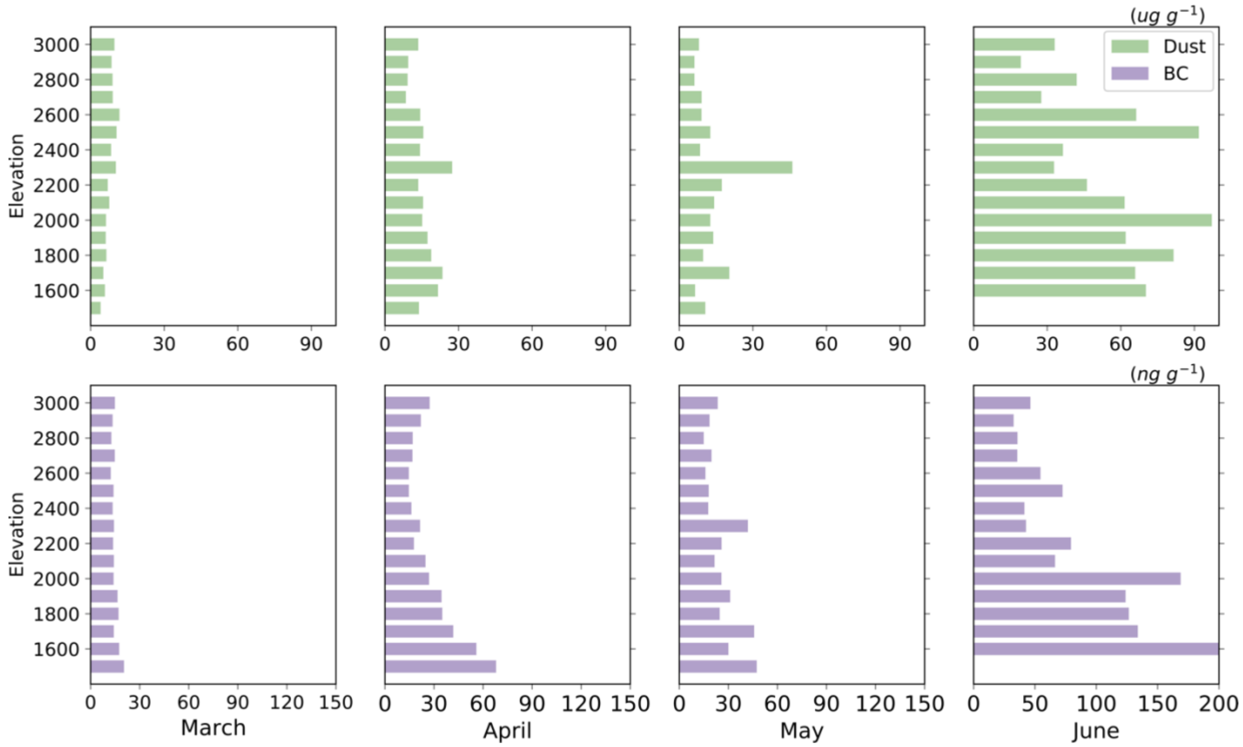

Figure S2 Concentration of dust (μg g^{-1}) and BC concentrations (ng g^{-1}) in the top snow layer (top 2 cm) at different elevation bands in WRF-Chem

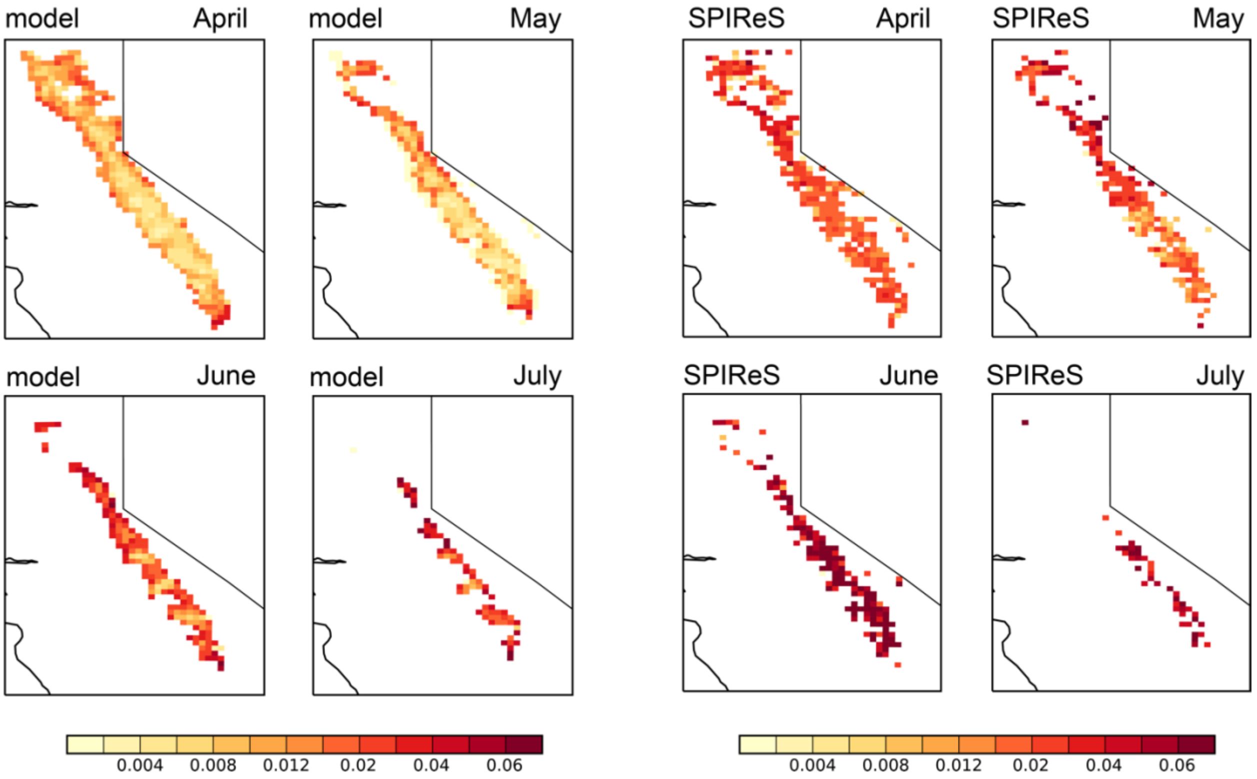

Figure S3 Monthly Mean snow albedo reduction ($\Delta\alpha$) from $WCCS_{aero}$ (excludes snow albedo feedbacks) and SPIReS.

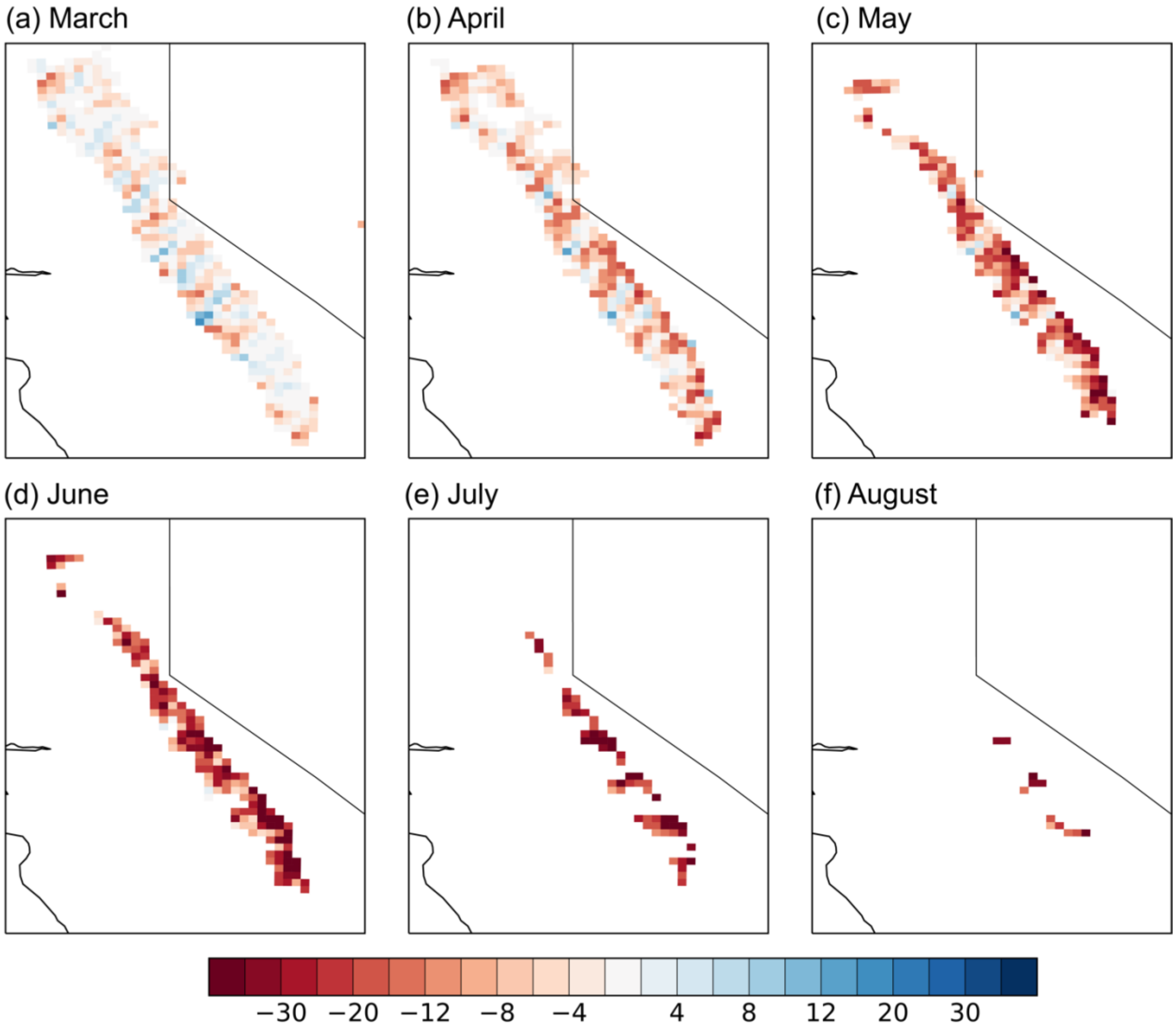

Figure S4 Relative changes of SWE (%) during March to August between $WCCS_{aero}$ and $WCCS_{noaero}$

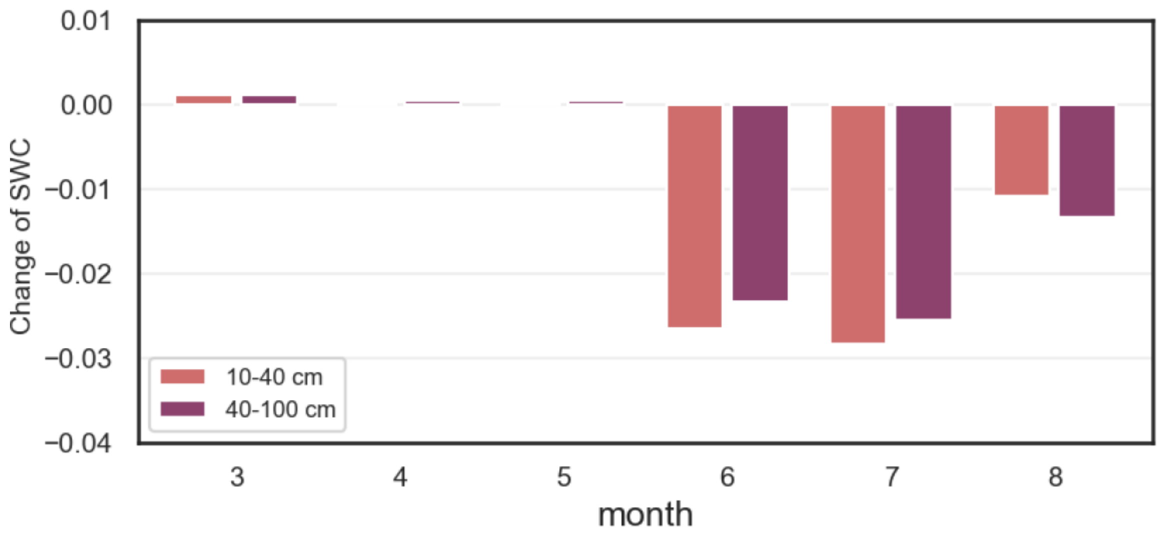

Figure S5 Change of soil water content between $WCCS_{aero}$ and $WCCS_{noaero}$ at 10-40 cm and 40-100 cm soil layers during March-August

SI References

Buchholz, R., L. Emmons, and S. Tilmes (2019), CESM2. 1/CAM-chem instantaneous output for boundary conditions, in *UCAR/NCAR-Atmospheric Chemistry Observations and Modeling Laboratory*, edited.

Chapman, E. G., W. I. Gustafson, R. C. Easter, J. C. Barnard, S. J. Ghan, M. S. Pekour, and J. D. Fast (2009), Coupling aerosol-cloud-radiative processes in the WRF-Chem model: Investigating the radiative impact of elevated point sources, *Atmos Chem Phys*, *9*(3), 945-964.

Easter, R. C., S. J. Ghan, Y. Zhang, R. D. Saylor, E. G. Chapman, N. S. Laulainen, H. Abdul-Razzak, L. R. Leung, X. D. Bian, and R. A. Zaveri (2004), MIRAGE: Model description and evaluation of aerosols and trace gases, *J Geophys Res-Atmos*, *109*(D20).

Fahey, K. M., and S. N. Pandis (2001), Optimizing model performance: variable size resolution in cloud chemistry modeling, *Atmos Environ*, *35*(26), 4471-4478.

Ginoux, P., M. Chin, I. Tegen, J. M. Prospero, B. Holben, O. Dubovik, and S. J. Lin (2001), Sources and distributions of dust aerosols simulated with the GOCART model, *J Geophys Res-Atmos*, *106*(D17), 20255-20273.

Kumar, R., M. Barth, G. Pfister, M. Naja, and G. Brasseur (2014), WRF-Chem simulations of a typical pre-monsoon dust storm in northern India: influences on aerosol optical properties and radiation budget, *Atmos Chem Phys*, *14*(5), 2431-2446.

Zhao, C., X. Liu, L. R. Leung, B. Johnson, S. A. McFarlane, W. I. Gustafson, J. D. Fast, and R. Easter (2010), The spatial distribution of mineral dust and its shortwave radiative forcing over North Africa: modeling sensitivities to dust emissions and aerosol size treatments, *Atmos Chem Phys*, *10*(18), 8821-8838.

Zhao, C., et al. (2014), Simulating black carbon and dust and their radiative forcing in seasonal snow: a case study over North China with field campaign measurements, *Atmos Chem Phys*, *14*(20), 11475-11491.
